# Efficacy of Oxygen–Ozone Therapy for Shoulder Disorders: A Systematic Review of Randomized Controlled Trials

**DOI:** 10.3390/jcm15145391

**Published:** 2026-07-09

**Authors:** Alessandro de Sire, Andrea Demeco, Andrea Racinelli, Francesco Agostini, Angelica Balena, Kristian Efremov, Umile Giuseppe Longo, Marco Invernizzi, Nicola Marotta, Antonio Ammendolia

**Affiliations:** 1 Physical and Rehabilitative Medicine, Department of Medical and Surgical Sciences, University of Catanzaro “Magna Graecia”, 88100 Catanzaro, Italy; alessandro.desire@unicz.it (A.d.S.); andrea.demeco@unicz.it (A.D.); andrea.racinelli@studenti.unicz.it (A.R.); angelica.balena@studenti.unicz.it (A.B.); ammendolia@unicz.it (A.A.); 2 Research Center on Musculoskeletal Health, MusculoSkeletalHealth@UMG, University of Catanzaro “Magna Graecia”, 88100 Catanzaro, Italy; 3 Department of Anatomical and Histological Sciences, Legal Medicine and Orthopedics Sapienza University, 00185 Rome, Italy; francesco.agostini@uniroma1.it; 4 Department of Orthopaedic Surgery, New York University-Langone Winthrop Hospital, Mineola, NY 11501, USA; 5 Fondazione Policlinico Universitario Campus Bio-Medico, Unit of Orthopaedic and Trauma Surgery, Department of Medicine and Surgery, Via Alvaro del Portillo, 200, 00128 Roma, Italy; g.longo@policlinicocampus.it; 6 Research Unit of Orthopaedic and Trauma Surgery, Department of Medicine and Surgery, Università Campus Bio-Medico di Roma, Via Alvaro del Portillo, 21, 00128 Roma, Italy; 7 Physical and Rehabilitative Medicine, Department of Health Sciences, University of Eastern Piedmont “A. Avogadro”, 28100 Novara, Italy; marco.invernizzi@med.uniupo.it; 8 Dipartimento Attività Integrate Ricerca e Innovazione (DAIRI), Translational Medicine, Azienda Ospedaliera SS. Antonio e Biagio e Cesare Arrigo, 15121 Alessandria, Italy; 9 Physical and Rehabilitative Medicine, Department of Experimental and Clinical Medicine, University of Catanzaro “Magna Graecia”, 88100 Catanzaro, Italy

**Keywords:** oxygen–ozone injections, shoulder disorders, corticosteroid injections, pain management, rehabilitation

## Abstract

**Background:** Shoulder disorders are among the leading causes of musculoskeletal pain and disability worldwide. Despite the widespread use of corticosteroid injections, concerns regarding their short-term efficacy and potential adverse effects have encouraged research into alternative therapies such as oxygen–ozone therapy. This systematic review aimed to evaluate the efficacy of oxygen–ozone therapy in the management of shoulder disorders. **Methods:** A systematic search of PubMed, Scopus, and Web of Science was conducted up to 16 March 2026, according to PRISMA guidelines. Randomized controlled trials investigating localized oxygen–ozone injections for shoulder disorders were included. Pain scores and shoulder function were the main outcomes considered. Study quality was assessed using the Cochrane Risk of Bias 2 tool. **Results:** Five randomized controlled trials involving 263 participants with shoulder impingement syndrome, adhesive capsulitis, or chronic rotator cuff syndrome were included. Given the heterogeneity of the included studies, we only performed a qualitative synthesis. Oxygen–ozone therapy demonstrated significant improvement in pain and shoulder function across all of the included studies. Corticosteroid injections generally showed superior short-term pain relief, whereas oxygen–ozone therapy demonstrated more persistent clinical improvements during medium-to-long follow-up periods. Studies employing multiple ozone injection protocols reported more stable outcomes when compared with single-injection approaches. No major adverse events related to oxygen–ozone therapy were reported. However, substantial heterogeneity was observed regarding injection protocols, ozone concentrations, number of injections, and follow-up duration. **Conclusions:** Oxygen–ozone therapy appeared to be a potentially effective treatment option for shoulder disorders, particularly in patients with contraindications to corticosteroids or chronic degenerative conditions. While corticosteroid injection might provide short-term symptom relief, repeated oxygen–ozone injection protocols seemed to offer sustained clinical benefits over time. Further high-quality randomized controlled trials with standardized protocols and longer follow-ups are needed to better define the role of oxygen–ozone therapy in shoulder rehabilitation.

## 1. Introduction

Shoulder disorders represent a leading cause of musculoskeletal pain and disability worldwide and are associated with a significant socioeconomic burden. Shoulder pain is among the most frequent musculoskeletal disorders in clinical practice, ranking third after low back and knee pain [1,2]. Epidemiological data indicate a high prevalence of shoulder pain in the general population, ranging from 0.67 to 55.2% over 12 months, and the incidence ranged from 7.7 to 62 per 1000 persons per year [3].

Recent Global Burden of Disease analyses have further highlighted the growing global impact of shoulder-related disorders, confirming their increasing contribution to disability and healthcare burden worldwide [4]. These conditions are strongly associated with reduced upper limb function, work limitations, and impaired quality of life; in particular, functional impairments frequently translate into limitations in activities of daily living, reduced participation in work and recreational activities, and long-term disability, which represent key targets of rehabilitative interventions [4].

Shoulder pain is a heterogeneous condition encompassing multiple disorders involving soft tissues, tendons, and joints [5]. Among these, shoulder impingement syndrome (SIS) is the most diagnosed clinical entity, accounting for a large proportion of shoulder-related medical consultations [6]. SIS results from the mechanical and functional narrowing of the subacromial space, leading to compression and irritation of the rotator cuff tendons, particularly the supraspinatus; the condition is multifactorial, involving intrinsic tendon degeneration and bursitis, as well as extrinsic biomechanical alterations, ultimately resulting in pain and functional limitation [6,7].

Rotator cuff-related disorders, including tendinopathy and structural tears, are also a major source of shoulder pain and disability [2]. Another relevant condition is rotator cuff calcific tendinopathy, characterized by calcium hydroxyapatite deposits within the tendon and predominantly affecting individuals between 30 and 60 years of age, leading to acute or persistent pain and functional impairment [7].

Moreover, adhesive capsulitis is a disabling condition characterized by progressive pain and a marked reduction in both active and passive range of motion (ROM), in the absence of significant structural abnormalities on imaging [8,9]. The condition is associated with capsular fibrosis and chronic inflammatory changes, resulting in significant restriction of shoulder mobility, particularly in abduction and external rotation [10].

Lastly, glenohumeral osteoarthritis is an increasingly more frequent degenerative condition that contributes substantially to shoulder disability, affecting up to 20% of the population and leading to significant, progressively worsening pain and functional decline [11].

Despite the wide range of available therapeutic options, including pharmacological treatments, physical therapy, corticosteroid injections, and surgery, clinical outcomes remain inconsistent, and a relevant proportion of patients experience persistent symptoms or incomplete recovery [12,13,14].

Although conservative management strategies such as physical therapy and electrotherapy are widely used, current evidence suggests variable and often limited clinical effectiveness, particularly in chronic presentations [12,15]. In addition to rehabilitation approaches, injection-based therapies, including corticosteroids, hyaluronic acid, and platelet-rich plasma, have been widely investigated in rotator cuff-related disorders, with evidence suggesting short-term improvements in pain and function [7]; however, results remain heterogeneous [13]. These findings highlight the need for alternative or adjunctive therapeutic strategies targeting the underlying inflammatory and degenerative processes.

In this context, the oxygen–ozone therapy has gained increasing interest in musculoskeletal rehabilitation. This technique involves the administration of a medical gas mixture composed of oxygen and ozone, which is proposed to exert anti-inflammatory, analgesic, and antioxidative effects [16,17]. At the molecular level, the proposed mechanism for oxygen–ozone therapy stands on the ability to modulate pro-inflammatory cytokines, improve tissue oxygen metabolism, and activate endogenous antioxidant pathways, thereby promoting tissue repair and modulating inflammation [16,18]. Clinical studies and systematic reviews have demonstrated its efficacy in reducing pain and improving function in conditions such as lumbar disk herniation and cervicobrachial pain [19,20,21], while broader evidence suggests potential benefits in tendinopathies and other musculoskeletal pathologies through modulation of inflammatory pathways and tissue healing processes [17,22,23]. However, despite these promising findings, evidence regarding the efficacy of oxygen–ozone therapy for shoulder disorders remains limited and fragmented. A recent scoping review analyzed the effectiveness of oxygen–ozone therapy for upper limb disorders, demonstrating encouraging results in terms of pain and functioning. Despite these findings, a more rigorous approach, focusing on RCTs, with a critical quality assessment and a more homogeneous PICO framework, is needed to better define the potential role of oxygen–ozone therapy, particularly for shoulder disorders [24]. Available studies are heterogeneous in terms of patient populations, intervention protocols, and outcome measures, and no comprehensive synthesis has yet critically evaluated its role across the spectrum of shoulder disorders.

Therefore, this systematic review aimed to critically evaluate the available evidence on the efficacy of oxygen–ozone therapy in the management of shoulder disorders, providing an updated and comprehensive synthesis to inform clinical practice and guide future research.

## 2. Materials and Methods

We searched PubMed, Scopus, and Web of Science for papers written in the English language published until 16 March 2026, according to a specific thesaurus, following the search strategy which is available in Table 1.

This systematic review was conducted according to the Preferred Reporting Items for Systematic Review and Meta-analysis (PRISMA) guidelines; the PRISMA checklist is available in the Supplementary Materials [25]. This systematic review protocol is available on the International Prospective Register of Systematic Reviews (PROSPERO), registration no.: CRD420261342221.

After removing duplicates, two reviewers (A.R., A.B.) independently screened all papers for title, abstract, and full text for eligibility. In case of disagreement, a third reviewer (A.d.S.) was consulted to allow consensus. All randomized controlled trials (RCTs) were assessed according to the following PICO (Population, Intervention, Comparison, and Outcomes) framework:

P, participants: adults affected with shoulder disorders causing pain and functional limitation;

I, intervention: oxygen–ozone injections in the shoulder anatomical district (intra-bursal, intra-articular, intra- or peri-tendinous, etc.);

C, comparison: any conservative intervention provided for shoulder disorders or sham treatment or placebo;

O, outcomes: pain measured through scales such as numerical rating scale (NRS) or Visual Analog Scale (VAS); additional outcome was functioning measured through scales such as SPADI (Shoulder Pain and Disability Index) or Constant–Murley Score (CMS).

The review was designed to evaluate the available evidence regarding the clinical effects of oxygen–ozone therapy in shoulder disorders. It was not intended to assess non-inferiority, equivalence, or superiority of oxygen–ozone therapy compared with any specific comparator intervention.

We included only RCTs that provided data at the end of the intervention. Our exclusion criteria were: (1) patients aged <18 years; (2) any RCT comparing surgical intervention; (3) studies with cross-over design; (4) studies written in any language other than English; (5) studies where full text was unavailable (i.e., posters or conference abstracts); (6) studies involving animal experimentation.

Two reviewers (A.R., A.B.) independently extracted data using a customized Microsoft Excel sheet from the included studies. In case of disagreement, a third reviewer (A.d.S.) was consulted to achieve consensus. We extracted the following data: (1) first author; (2) publication year; (3) nationality; (4) study participants; (5) comparison; (6) type of intervention; (7) outcome measures; (8) pathology-treated (9) main findings. Data extracted from the included studies are available on request.

The selected studies were analyzed by using the Cochrane Risk of Bias tool for Randomized Controlled Trials (RoB 2, Version 22 August 2019) tool [26]. Two reviewers (A.R., A.B.) independently assessed the study quality; in case of disagreement, a third reviewer (A.d.S.) was asked to resolve conflicts to allow consensus. According to the RoB 2 [26], risk of bias was defined as “low risk”, “some concerns”, and “high risk”.

Moreover, the certainty of evidence for the primary outcome was assessed using the Grading of Recommendations Assessment, Development and Evaluation (GRADE) approach. Certainty was evaluated across the domains of risk of bias, inconsistency, indirectness, imprecision, and publication bias, and was rated as high, moderate, low, or very low [27].

## 3. Results

**Figure 1:**
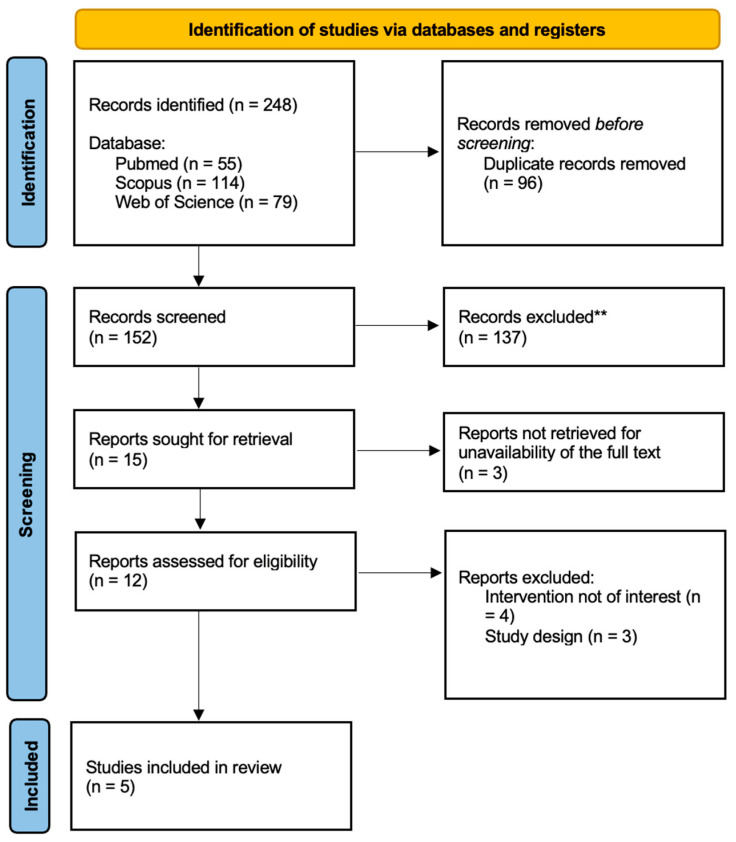
PRISMA flow diagram. (** no automation tool was used).

A total of 248 articles were found in the initial search; after removing duplicates, 152 reports were screened by title and abstract. A total of 137 records were excluded. The remaining 15 reports were assessed for eligibility according to the PICO framework; three of them were not retrieved because the full text was unavailable. We excluded four reports because the intervention was not of interest; three were excluded because the study design differed from an RCT. We finally included five RCTs [28,29,30,31,32] in this systematic review (see Figure 1 for further details).

The included studies were published in 2019 [28], 2023 [29,31], and 2025 [30,32]. Three of them were published in Turkey [29,30,32], one was published in Iran [28], and one was published in Egypt [31]. The overall number of study participants was 263, of which 159 were females (60.5%), and 104 were males (39.5%). Participants were predominantly middle-aged, ranging from 30 to 70 years old. Among the included studies, two studies [30,31] analyzed patients diagnosed with adhesive capsulitis; two studies [28,32] analyzed patients diagnosed with shoulder impingement syndrome, and one study [29] analyzed patients diagnosed with chronic rotator cuff syndrome. The outcome assessor was blinded in each study, but none of the included studies were blinded for the patients or the physicians performing the intervention. The summarized characteristics of the included studies are available in Table 2. Given the impossibility of performing a meta-analysis, we followed Synthesis Without Meta-analysis (SWIM) guidelines [33] to correctly report the qualitative synthesis; the SWIM checklist is available in the Supplementary Materials.

### 3.1. Characteristics of Interventions

Most of the included studies [28,29,30] compared ozone injections with corticosteroid injections, while Foula et al. [31] compared ozone injections with corticosteroid injections and pulsed radiofrequency (PRF), and Turgut et al. [32] compared a single ozone injection with multiple ozone injections and corticosteroid injections. The interventions in the study groups differed substantially. Particularly, two studies [28,31] performed a single oxygen–ozone injection with different concentrations: 8 mL, 12 μg/mL [28], and 10 mL, 15 μg/mL [31]. Two studies [29,30] performed multiple oxygen–ozone injections. Specifically, Atar et al. [29] performed three sessions administered once a week with subacromial bursa injections of 5 mL of oxygen–ozone mixture with different concentrations per session (10 μg/mL, 15 μg/mL, and 20 μg/mL) [29]; Sahillioglu et al. [30] performed eight sessions twice a week with intra-articular injections of 20 mL at a concentration of 15 μg/mL [30]. Finally, Turgut et al. [32] compared single and multiple oxygen–ozone injections; both groups were injected with 5 mL at a concentration of 10 μg/mL. In the multiple injections group, five sessions were performed once per week [32].

### 3.2. Characteristics of the Outcomes

The primary outcome analyzed was the efficacy of oxygen–ozone therapy on pain measured through the VAS. Three studies [28,31,32], comparing a single oxygen–ozone injection with a single corticosteroid injection, demonstrated a significant reduction in pain for both groups. Particularly, Babaei-Ghazani et al. [28] observed a stronger reduction in VAS for the corticosteroid group at two weeks from the intervention (ΔVAS T0 − T1 steroid group = 4.80; ΔVAS T0 − T1 ozone group = 2.00, both *p* < 0.05) while at two months from the intervention, there was a slight increase in VAS for the corticosteroid group compared with T1 (ΔVAS T1 − T2 steroid group = −0.33, *p* > 0.05) with a maintained reduction for the ozone group (ΔVAS T − T2 ozone group = 0.68 *p* < 0.05). No statistically significant difference was observed between the two groups at any timepoint [28]. Foula et al. [31] measured both the Visual Analog Scale at rest (VASr) and during movement (VASm) of the shoulder. For each group, there was a significant reduction in VAS values at week one, two, and four throughout the follow-up (*p* < 0.001), while at week eight, a third group treated with pulsed radiofrequency showed a significant difference compared with the ozone group and the corticosteroid group (*p* = 0.024 and *p* = 0.01 for VASm and VASr) [31]. Finally, Turgut et al. [32] was the only study analyzing patients with a 1-year follow-up. The reduction in VAS was statistically significant among all study groups at T1 (1 week after the end of the intervention protocol), but the corticosteroid group improved significantly more (*p* < 0.001). At 1-year follow-up, the only group in which the improvement in VAS remained stable was the multiple oxygen–ozone therapy injection group (T3 = median 4.0; IQR 1.0 in the multiple ozone injection group vs. T3 = median 7; IQR 2.0 in the single ozone injection group and T3 = median 7; IQR 0.0 in the corticosteroid group; *p* < 0.001) [32]. Studies comparing multiple ozone injections with corticosteroids showed a significant reduction throughout the follow-up period [29,30]. In particular, Atar et al. [29] demonstrated a significant improvement in VAS at 1- and 3-month follow-ups in both groups (*p* < 0.001), with no significant difference comparing the corticosteroid and ozone groups (Time x group *p* = 0.146) [29]. Sahillioglu et al. [30] observed significant improvement in VAS at night and during motion, both at 1 month and 3 months, for both groups (*p* < 0.001) with no significant difference between the two groups (*p* > 0.05) [30].

To facilitate interpretation of the clinical heterogeneity among the included studies, pain outcomes were additionally stratified according to the underlying shoulder disorder. A diagnosis-specific synthesis of the findings is reported in Table 3.

The secondary outcome was shoulder function. In detail, there was a significant heterogeneity among the included studies regarding the scales used to assess functioning. The most commonly used scale was SPADI [28,29,30,31]. In each study, a significant improvement in SPADI scores was observed. Babaei-Ghazani et al. [28] observed a more sustained improvement in the corticosteroid group; this observation was associated with the same improvement in Constant Score, especially two months after the intervention [28]. This finding differs from the other included studies in which SPADI improved in the ozone groups (*p* < 0.001 for each statistical analysis), with no significant difference from the other interventions at any timepoint throughout the follow-up assessments [29,30,31]. The findings regarding shoulder function observed by Atar et al. [29] are also consistent when evaluated with the Western Ontario Rotator Cuff Index (WORC), with an improvement in the study group (*p* < 0.001) and no difference in the control group (*p* = 0.071) [29]. The Constant Score was also assessed by Turgut et al. [32], with a significant improvement in both groups, with more favorable results in the multiple ozone injection group at the 1-year follow-up (*p* < 0.001). This finding was in line with the University of California Los Angeles Shoulder Scale (UCLA shoulder scale), with the same observation throughout the follow-up [32].

Three studies measured the shoulder range of motion (ROM) [28,30,31]. The findings were similar for each study, with an improvement among the groups and no significant between-group difference [28,30,31]. Among the included studies, no paper reported any adverse effect [28,29,30,31,32].

### 3.3. Quality Assessment

We also performed a quality assessment according to Cochrane RoB-2 [26]. The most critical concern involved a possible bias related to the impracticality of blinding the patients and the physician performing the intervention because of the different kinds of injection (mostly liquid vs. gas or PRF). Therefore, the primary methodological limitation, consistently observed across all studies, is intrinsically linked to deviations from the intended interventions (D2). Specifically, the inherent nature of the comparative treatments, involving the administration of substances with radically different physical properties, such as liquids (e.g., corticosteroids), gases (oxygen–ozone therapy), or physical modalities (pulsed radiofrequency), precluded the feasibility of implementing a strict double-blind protocol. One study showed some concerns regarding the randomization process (D1), as the protocol to conceal the randomization was not clearly stated [30].

**Figure 2:**
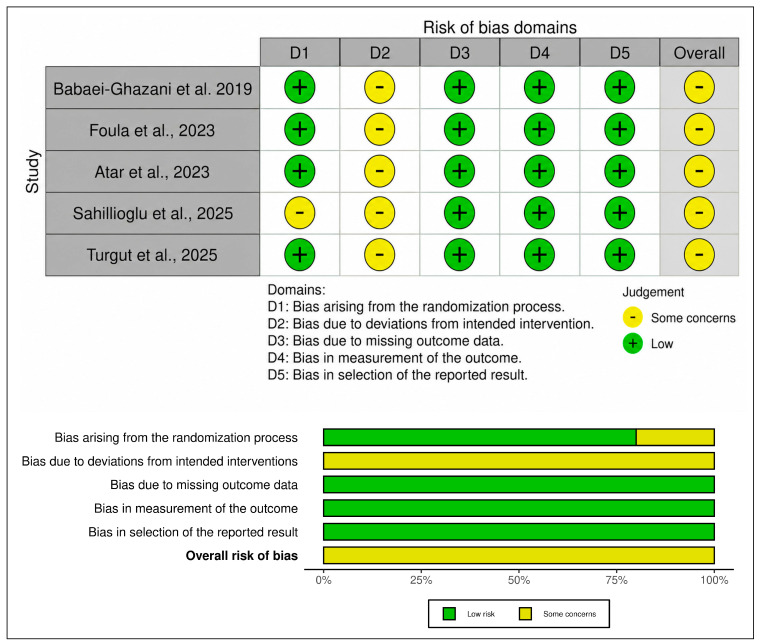
Quality assessment [26,28,29,30,31,32].

The other domains did not report any significant risk of bias [28,29,30,31,32]; optimal validity was observed across all studies regarding missing outcome data (D3), the measurement of clinical outcomes (D4), and the selection of the reported results (D5), thereby confirming the strong internal validity of the clinical trials included in this review. More details are available in Figure 2.

### 3.4. Summary of Findings

The certainty of evidence for the primary outcome (pain assessed through the Visual Analog Scale [VAS]) was evaluated using the GRADE framework [27]. Overall, the certainty of evidence was judged as low (⊕⊕◯◯). The certainty was downgraded by one level for risk of bias, as all included studies were randomized controlled trials, and none was judged at high risk of bias according to the RoB2 assessment. Although all studies presented some concerns, these were mainly related to deviations from intended interventions resulting from the inherent difficulties in blinding participants and clinicians during injection-based procedures. No downgrading was applied for inconsistency because all studies consistently reported pain improvement following oxygen–ozone treatment, despite variations in treatment protocols and comparator interventions. Similarly, no concerns regarding indirectness were identified, as the populations, interventions, comparators, and outcomes were consistent with the review PICO. Publication bias was not suspected. The certainty of evidence was downgraded by one more level for imprecision because the available evidence was derived from a limited number of relatively small randomized controlled trials, and no pooled quantitative estimate was available. The full summary of findings is available in Table 4.

## 4. Discussion

This systematic review aimed to critically synthesize the available literature regarding the efficacy of oxygen–ozone therapy in shoulder disorders. The included studies investigated different shoulder disorders characterized by distinct pathophysiological mechanisms and clinical trajectories. Therefore, the findings should not be interpreted as evidence of a uniform treatment effect across all shoulder conditions. Rather, the available evidence suggests that oxygen–ozone therapy may provide clinical benefits across different shoulder disorders, although the magnitude and temporal profile of these effects appear to vary according to the underlying diagnosis and treatment protocol.

Our most consistent finding was the effect on pain measured through VAS. It is interesting to note that across the included studies, oxygen–ozone therapy was associated with clinically meaningful improvements in pain and function. Although several trials reported no statistically significant between-group differences when compared with corticosteroid injections, the available evidence does not allow conclusions regarding superiority, non-inferiority, or equivalence between interventions, but the corticosteroids appeared more effective in short-term pain reduction. At the same time, in longer follow-ups, oxygen–ozone therapy appeared to report a more persistent clinical improvement [28,32]. These findings suggest that oxygen–ozone therapy could be an effective intervention for shoulder disorders, particularly in patients for whom corticosteroids cannot be selected due to personal clinical history or contraindications. For example, in diabetic patients, transitory hyperglycemia was noted after a subacromial corticosteroid injection lasting for two days after the procedure [34]. Additionally, it is well known that corticosteroids can have a long-term detrimental effect on both cartilage and tendons, while oxygen–ozone therapy does not seem to have any significant long-term consequences [35].

It is crucial to emphasize the difference between the two approaches, even for different kinds of procedures (whether subacromial or intra-articular) and different disorders (adhesive capsulitis, rotator cuff syndrome, and shoulder impingement syndrome), the short-term effect of corticosteroid injections was considerably higher than that of ozone in most of the included studies [28,32]. These findings are in line with the existing literature concerning the comparison between oxygen–ozone and corticosteroid injections. Particularly, a previous multicenter RCT conducted by Aslan et al. in 2024 [36] showed a significant improvement in pain, consistent through time for both groups of patients affected with knee osteoarthritis treated with oxygen–ozone and corticosteroid injections [36]. Besides the clinical efficacy of oxygen–ozone and corticosteroid injections, the different pattern in terms of response profile is probably associated with the different dynamics of the substances injected. Our observations concerning the possibility of a slow and long-term efficacy of oxygen–ozone therapy, whether still confirmed by a small amount of data, which limits the possibility of stronger conclusions [28,29,30,31,32], are coherent with its mechanism of action; namely, shoulder disorders are correlated with chronic inflammation; in fact, there is a consistent literature regarding the increase in pro-inflammatory cytokines, such as IL-1, IL-6, and metalloproteases in chronic tendinopathies and osteoarthritis [37,38,39]. In this context, ozone was proven effective as an anti-inflammatory therapy; the interaction of ozone with the water in the tissues leads to the formation of hydrogen peroxide, which mediates the effects at a cellular level, contributing to the increase in transcription factors and leading to the activation of antioxidant enzymes. This dynamic could lead to a biological, long-term anti-inflammatory effect and, at the same time, fibroblast proliferation and tissue remodeling [37,38,39]. Another crucial aspect of our findings is the difference between a single and multiple injections of the oxygen–ozone mixture. In the studies comparing multiple injections with a single corticosteroid injection [29,30], the effects of the oxygen–ozone therapy appeared to be more stable over time, with no significant difference compared to the corticosteroid group. This is an interesting finding because a structured cycle could overcome the higher anti-inflammatory impact of corticosteroid injections in the short- and mid-term. On the other hand, the need for more sessions could reduce the compliance of the patient. It is crucial to emphasize that this is still an initial comparison with limited data, thus limiting the generalizability. Only one study compared both single and multiple injections [32]. This study, while showing an initial improvement more consistent in the corticosteroid group, demonstrated that multiple ozone injections could lead to long-term benefits for the patients. Despite these findings on the possible long-term efficacy of oxygen–ozone therapy, the evidence is still limited and should be seen as a preliminary result worthy of further analysis. In this context, it is important to emphasize that the actual recommendations for oxygen–ozone therapy from the International Scientific Committee of Ozone Therapy 3 (ISCO3) recommend a multiple-injection approach with an increasing dosage throughout the sessions [40].

In this scenario, the multiple-injection protocols could have a cumulative effect, with repeated biological stimulation of the tissues. In shoulder disorders associated with chronic inflammation and degeneration, the possibility of increasing biological stimuli could reduce these processes, thus giving the possibility of tissue healing. This evidence was observed by Foula et al. [31], who demonstrated a significant reduction in two biological markers (ICAM-1 and hs-CRP) at eight weeks from the intervention [31]. Thus, the variability of outcomes among the included studies may partially depend on differences in ozone administration protocols, particularly the number of treatment sessions and the concentration used to inject. Studies employing repeated ozone injections generally reported more durable clinical improvements compared with single-injection protocols, suggesting that the therapeutic effects of oxygen–ozone therapy may be cumulative and biologically progressive rather than immediate. Despite these findings, the evidence base remains limited; thus, definitive conclusions should be cautious. Our findings suggest that oxygen–ozone therapy could be a valid approach, especially for patients affected by corticosteroid contraindications (i.e., diabetes) or degenerative tendinopathies. At the same time, more standardized protocols should be developed in order to obtain clearer guidelines for proposing oxygen–ozone therapy in the field of shoulder disorders.

Moreover, oxygen–ozone therapy for shoulder disorders could represent a valid approach, as among the included studies, no major adverse event was reported. However, the absence of a clear method to report adverse events, the small sample sizes, and the short follow-ups currently limit conclusions concerning safety. Moreover, some limitations should be acknowledged, particularly associated with the need for multiple treatment sessions, which may increase treatment burden and healthcare utilization, and the heterogeneity regarding the protocol of injections.

Despite its methodological strengths and the clinical relevance of its findings, this systematic review is not free of limitations. Firstly, a quantitative meta-analysis could not be performed due to the substantial heterogeneity among the included trials, which varied significantly regarding injection protocols, ozone concentrations, the total number of treatment sessions, anatomical injection sites, pathology treated, and follow-up durations. Secondly, the coexisting home-based physical rehabilitation programs were not standardized across the selected studies, introducing a confounding variable that limits the clear interpretability of the clinical outcomes. Thirdly, from a statistical perspective, the included evidence is characterized by relatively small sample sizes, encompassing an overall cohort of only 263 participants across five trials, which may restrict the broader generalizability of the conclusions. Fourthly, a notable methodological constraint was the impossibility of blinding both the patients and the treating physicians due to the radically different physical properties of the compared interventions (e.g., gas versus liquid), thereby introducing a potential risk of performance bias; particularly, the possibility of expectation, performance, and placebo effects cannot be excluded. This aspect is particularly relevant because the primary and secondary outcomes assessed across the included studies were largely patient-reported measures, including pain intensity and functional status. Therefore, part of the observed clinical improvement may have been influenced by contextual and non-specific treatment effects rather than exclusively reflecting the biological effects of oxygen–ozone therapy. Lastly, the complete absence of placebo- or sham-controlled trials among the included literature precludes a definitive determination of the extent to which the observed functional and analgesic improvements are directly attributable to the biological properties of the oxygen–ozone therapy rather than to contextual or placebo-related factors. However, despite these limitations, a consistent trend was observed, particularly regarding pain reduction and functional improvement following oxygen–ozone therapy. There is a need for future studies to focus on the development of standardized treatment protocols, particularly regarding ozone concentration, injected volume, number of sessions, and injection site, with longer follow-up periods, possibly specifying the concurrent rehabilitation protocol performed. Moreover, further studies should ideally be larger randomized controlled trials, with prespecified minimal clinically important differences, adverse-event monitoring, and stratification by shoulder diagnosis.

## 5. Conclusions

In conclusion, the current systematic review suggested that oxygen–ozone therapy could be an effective intervention for the management of shoulder disorders, with limited adverse events, leading to improvements in pain and shoulder function across different clinical conditions. While corticosteroid injections could provide faster short-term pain relief, oxygen–ozone therapy administered through repeated injection protocols might offer more sustained clinical benefits over time, with fewer side effects and contraindications. These observations are still limited and need to be confirmed with further studies. Moreover, the variability among the available studies, especially regarding injection protocols and follow-up duration, currently might limit the possibility of establishing definitive recommendations. Nevertheless, the oxygen–ozone therapy may represent a valuable therapeutic option, particularly in patients with contraindications to corticosteroids or chronic degenerative shoulder conditions. Further high-quality randomized controlled trials with standardized protocols and longer follow-up periods are needed to better define the role of oxygen–ozone therapy in shoulder rehabilitation.

## Figures and Tables

**Table 1:** Search strategy.

**Pubmed:** (“ozone” [Mesh] OR ozone OR “oxygen ozone” OR “oxygen-ozone” OR “O_2_–O_3_”) AND (shoulder OR “rotator cuff” OR supraspinatus OR “adhesive capsulitis” OR “frozen shoulder” OR bursitis OR impingement OR tendinopathy OR tendinitis OR “subacromial pain syndrome”)
**Scopus:** TITLE-ABS-KEY (“ozone” OR “oxygen ozone” OR “oxygen-ozone” OR “O_2_–O_3_”) AND (shoulder OR “rotator cuff” OR supraspinatus OR “adhesive capsulitis” OR “frozen shoulder” OR bursitis OR impingement OR tendinopathy OR tendinitis OR “subacromial pain syndrome”)
**Web of Science:** (ozone OR “oxygen ozone” OR “oxygen-ozone” OR “O_2_–O_3_”) AND (shoulder OR “rotator cuff” OR supraspinatus OR “adhesive capsulitis” OR “frozen shoulder” OR bursitis OR impingement OR tendinopathy OR tendinitis OR “subacromial pain syndrome”)

**Table 2:** Characteristics of the included studies.

Author and Year	Nation	Pathology Treated	Study Groups	Control	Intervention	Comparison	Outcomes Measured	Main Findings
**Babaei-Ghazani et al. (2019) [28]**	Iran	Shoulder Impingement Syndrome	Ozone injection group (*n* = 15)	Corticosteroid injection group (*n* = 15)	Ultrasound-guided single injection into the subacromial bursa: • Ozone (O_2_–O_3_) injection (8 mL, 12 μg/mL) • Lidocaine 1% (2 mL) • Daily home exercise program (ROM, stretching, isometric exercises)	Ultrasound-guided single injection into the subacromial bursa: • Corticosteroid (triamcinolone 40 mg/mL, 1 mL) + lidocaine 1% (2 mL); • Same home exercise program; • No additional interventions.	Primary: VAS, Constant Score, SPADI Secondary: range of motion, ultrasound parameters (subacromial bursa thickness, supraspinatus tendon thickness, acromiohumeral distance)	Both groups showed significant improvements in pain and function; corticosteroid injection resulted in greater short-term improvement while ozone demonstrated a progressive improvement over time.
**Atar et al. (2023) [29]**	Turkey	Chronic Rotator Cuff Syndrome	Ozone injection group (*n* = 20)	Corticosteroid injection group (*n* = 20)	Ultrasound-guided subacromial injections (3 sessions, 1/week): • Ozone (O_2_–O_3_) injection (5 mL per session) • Increasing concentrations: 10, 15, and 20 μg/mL • Daily home exercise program (ROM, stretching, strengthening)	Ultrasound-guided subacromial injection: • Corticosteroid (betamethasone 3 mg/mL, 1 mL) + lidocaine (20 mg, 1 mL); • Single injection; • Same home exercise program.	Primary: WORC (Western Ontario Rotator Cuff Index) Secondary: VAS, SPADI	Both groups showed significant improvements in pain, function, and quality of life; however, no statistically significant differences were found between ozone and corticosteroid injections groups.
**Sahillioğlu et al.** ** (2025) [30]**	Turkey	Adhesive Capsulitis	Ozone injection group (*n* = 20)	Corticosteroid injection group (*n* = 20)	Ultrasound-guided intra-articular injections (8 sessions, 2/week for 4 weeks): • Ozone (O_2_–O_3_) injection (20 cc, 15 μg/mL) • Standardized home-based exercise program (Codman exercises, active-assisted ROM, and capsular stretching), initiated 2 weeks after the first injection	Ultrasound-guided intra-articular injection: • Corticosteroid (betamethasone 7 mg/1 mL); • Single injection; • Same standardized home-based exercise program.	Primary: VAS Secondary: SPADI, shoulder range of motion	Both groups showed significant improvements in pain, function, and range of motion; however, no statistically significant differences were found between ozone and corticosteroid injections groups.
**Foula et al.** **(2023) [31]**	Egypt	Adhesive Capsulitis	Ozone injection group (*n* = 15)	Corticosteroid group (n = 15). Pulsed radiofrequency groups (*n* = 15).	Ultrasound-guided intra-articular injection: • Ozone (O_2_–O_3_) injection (10 mL, 15 μg/mL) • Bupivacaine 0.125% (5 mL)	Ultrasound-guided intra-articular procedures: • Corticosteroid (triamcinolone 40 mg) + bupivacaine 0.125% (5 mL); • Intra-articular pulsed radiofrequency application (4 min).	Primary: VAS (rest and movement) Secondary: SPADI, shoulder range of motion, inflammatory markers (ICAM-1, hs-CRP)	All groups showed significant improvements in pain, function, shoulder range of motion, and inflammatory markers; corticosteroid provided faster short-term pain relief, while pulsed radiofrequency demonstrated superior long-term improvement, and ozone demonstrated intermediate improvements.
**Turgut et al.** **(2025) [32]**	Turkey	Shoulder Impingement Syndrome	Ozone injection groups single-dose (n = 36) and multiple-dose (*n* = 36)	Corticosteroid injection group (*n* = 36)	Ultrasound-guided subacromial injection: • Single-dose ozone (O_2_–O_3_) injection (5 mL, 10 μg/mL; total 50 μg) • Multiple-dose ozone (O_2_–O_3_) injections (5 mL, 10 μg/mL; once weekly for 5 weeks)	Ultrasound-guided subacromial injection: • Corticosteroid (triamcinolone 40 mg, 1 mL) + bupivacaine (5 mg, 1 mL); • Single injection; • Patients were instructed to avoid physiotherapy and NSAIDs during the study period.	Primary: VAS Secondary: Constant–Murley Score (CMS), UCLA Shoulder Scale, SF-36	All groups showed significant improvements in pain, function, and quality of life; corticosteroid injection provided faster short-term pain relief, whereas multiple-dose ozone demonstrated superior long-term outcomes compared to both single-dose ozone and corticosteroid injection.

Abbreviations: VAS: Visual Analog Scale; SPADI: Shoulder Pain and Disability Index; SF-36: Short-Form 36; WORC: Western Ontario Rotator Cuff Index.

**Table 3:** Stratified Synthesis of Pain Outcome According to Shoulder Disorder [28,29,30,31,32].

Pathology	Study	Comparator	Follow-Up	Main Findings on Pain
Shoulder Impingement Syndrome	Babaei-Ghazani 2019 [28]	Corticosteroid	2 months	Both groups improved; corticosteroid showed greater short-term improvement, while ozone demonstrated progressive improvement over time
Shoulder Impingement Syndrome	Turgut 2025 [32]	Corticosteroid; single vs. multiple ozone	1 year	Multiple ozone injections showed more stable long-term pain improvement
Adhesive Capsulitis	Foula 2023 [31]	Corticosteroid; PRF	8 weeks	Significant pain reduction in all groups; PRF superior at long-term follow-up
Adhesive Capsulitis	Sahillioğlu 2025 [30]	Corticosteroid	3 months	Significant pain reduction in both groups without between-group differences
Chronic Rotator Cuff Syndrome	Atar 2023 [29]	Corticosteroid	3 months	Significant pain improvement in both groups without significant between-group differences

**Table 4:** Summary of Findings.

Outcome	Studies	RoB	Inconsistency	Indirectness	Imprecision	Publication Bias	Certainty
Pain (VAS)	5 RCTs	Serious ^1^	Not serious ^2^	Not serious ^3^	Serious ^4^	Undetected	⊕⊕◯◯ Low

^1^ All studies were randomized controlled trials, and none was judged at high risk of bias, although we downgraded by one level because some concerns were observed due to the inability to fully blind participants and clinicians. ^2^ All studies reported pain improvement following oxygen–ozone treatment, with no conflicting direction of effect. ^3^ Included populations, interventions, comparators, and outcomes were consistent with the review PICO. ^4^ Downgraded by one level due to the relatively small sample sizes of the included studies and the absence of a pooled quantitative estimate. ⊕⊕◯◯ low certainty of findings.

## Data Availability

Data available on reasonable request.

## Supplementary Materials

The following supporting information can be downloaded at https://www.mdpi.com/article/10.3390/jcm15145391/s1, Table S1: PRISMA checklist [25].

## Abbreviations

The following abbreviations are used in this manuscript:

PRISMA	Preferred Reporting Items for Systematic Reviews and Meta-Analyses
SIS	Shoulder Impingement Syndrome
ROM	Range of Motion
PROSPERO	International Prospective Register of Systematic Reviews
RCT	Randomized Controlled Trial
PICO	Population, Intervention, Comparison, Outcomes
NRS	Numerical Rating Scale
VAS	Visual Analog Scale
SPADI	Shoulder Pain and Disability Index
RoB 2	Risk of Bias 2
O_2_–O_3_	Oxygen-Ozone
WORC	Western Ontario Rotator Cuff Index
ICAM-1	Intercellular Adhesion Molecule-1
hs-CRP	High-Sensitivity C-Reactive Protein
CMS	Constant–Murley Score
UCLA	University of California Los Angeles
SF-36	Short Form-36 Health Survey
VASr	Visual Analog Scale at Rest
VASm	Visual Analog Scale during Movement
IQR	Interquartile Range
PRF	Pulsed Radiofrequency
IL-1	Interleukin-1
IL-6	Interleukin-6
ISCO3	International Scientific Committee of Ozone Therapy
